# Supplementary material for: The Use of Drones to Deliver Rift Valley Fever Vaccines in Rwanda: Perceptions and Recommendations
Source: Vaccines (Basel). 2023 Mar 7;11(3):605. doi: 10.3390/vaccines11030605 (PMC10059257; doi:10.3390/vaccines11030605)
Supplement: Supplementary file 1 [file vaccines-11-00605-s001.zip › vaccines-2227754-supplementary.pdf]

Interview Guide

Veterinarians

1. *Job Description*
    a. What is your job title and job description*?*
    b. What is your involvement and experience in the livestock sector?
2. *Knowledge on RVF*
    a. One of the zoonosis that has impacted Nyagatare is RVF. What do you know about RVF? Is this a disease you have faced in your work field?
3. *Control and prevention of RVF*
    a. How have you gone about treating RVF infected livestock? What treatments and vaccines did you use?
    b. What was the procedure of acquiring RVF treatment and vaccine?
    c. Did you face any challenges in acquiring and administering treatment and vaccines?
4. *Opportunities for improvements/challenges*
    a. Taking these challenges into account, how can the prevention and control of RVF be improved?
5. *Knowledge and uses of UAVs in controlling and preventing RVF*
    a. What do you know about UAVs?
    b. In your view, would you use UAVs to help in RVF treatment and vaccine delivery?
    c. How big is the shipment per month you may need to effectively respond to RVF?
6. *Challenges and Opportunities for the use of drone*
    a. Do you anticipate any challenges with the use of UAVs in the delivery of RVF treatment and vaccines?
    b. What are they and how can they be resolved?
7. Policy questions
    a. Is there any policy that may enhance or inhibit the extension of drone use to livestock sector
    b. How can your organization help to facilitate UAV policy implementation in animal sector?

ZIPLINE STAFF

I. Company structure and operation
    a. What is your job title and job description at Zipline?
    b. How is the company structured?
    c. How are products currently delivered?
        i. Routes
        ii. Contract with hospitals
        iii. Cost
    d. What sectors (e.g. human, animal, environmental) does Zipline currently operate in and why?
        i. Do you have any future plans to expand beyond these sectors? Why or why not?

II. *Expansion to animal health sector*

a. Are there any veterinarians employed by zipline?
b. Would it be feasible to deliver vaccine and treatment for RVF to places like Nyagatare and other hard to reach areas?
c. What would be required to deliver vaccines (e.g. cold chain)?
d. What would be required to expand to the animal sector in terms of planning, marketing, contracts, etc?

III. *Challenges and Opportunities for the use of drone*

a. What has been the challenges in the delivery of human health products in hard to reach areas?
b. Will you anticipate the similar challenges in livestock sector?
c. How can these problems be solved according to your experience?
d. Besides RVF disease response, is there any other area you think of those UAVs can be used as an opportunity to boost livestock production in Rwanda?
